# Emerging role of long noncoding RNA-encoded micropeptides in cancer

**DOI:** 10.1186/s12935-020-01589-x

**Published:** 2020-10-16

**Authors:** Mujie Ye, Jingjing Zhang, Meng Wei, Baihui Liu, Kuiran Dong

**Affiliations:** 1 Department of Pediatric Surgery, Children’s Hospital of Fudan University, No.399 Wanyuan Road, Minhang District, Shanghai, 201102 China; 2 Key Laboratory of Neonatal Disease, Ministry of Health, Shanghai, 201102 China; 3 Department of Medical Imaging, Nanjing Hospital of Chinese Medicine Affiliated to Nanjing University of Chinese Medicine, Nanjing, 210001 China

**Keywords:** lncRNA, Open reading frame, Coding potential, Micropeptide, Cancer

## Abstract

Increasing evidence has indicated that long noncoding RNAs (lncRNAs) play various important roles in the development of cancers. The widespread applications of ribosome profiling and ribosome nascent chain complex sequencing revealed that some short open reading frames of lncRNAs have micropeptide-coding potential. The resulting micropeptides have been shown to participate in N6-methyladenosine modification, tumor angiogenesis, cancer metabolism, and signal transduction. This review summarizes current information regarding the reported roles of lncRNA-encoded micropeptides in cancer, and explores the potential clinical value of these micropeptides in the development of anti-cancer drugs and prognostic tumor biomarkers.

## Background

The mammalian genome produces huge numbers of transcripts during the transcription process; however, about 98% of human RNA transcripts are non-coding [1]. Non-coding RNAs can be classified into microRNAs (miRNAs), long noncoding RNAs (lncRNAs), circular RNAs (circRNAs), and small nucleolar RNAs (snoRNAs), all of which have different regulatory functions[2]. Among them, lncRNAs are a class of RNAs > 200 nucleotides long that do not code for proteins[3, 4]. Dysregulation of lncRNAs has been shown to be involved in physiological processes such as the regulation of gene expression [5], chromatin remodeling [6], maintenance of pluripotency[7], DNA damage repair [8], competing endogenous RNAs [9, 10], and it also can participate in some pathological processes such as tumorigenesis [11].

Developments in next-generation sequencing and advances in bioinformatics have revealed novel insights into the roles and functions of lncRNAs, including their potential to encode functional micropeptides [12]. Functional micropeptides are usually encoded by open reading frames (ORFs) in ncRNAs, including lncRNAs, circRNAs, and pre-miRNAs [13]. Current studies on lncRNA-encoded peptides in humans have mainly focused on their role in malignant tumors. These micropeptides serve as oncogenic drivers or tumor suppressors, similar to coding and noncoding genes, and play functional roles in processes including cancer onset, promotion, and progression by taking part in tumor angiogenesis, N6-methyladenosine (m6A) modification, signaling pathway transduction, and cancer metabolism[1, 14].

There are several reasons why previous studies may have failed to detect these peptides. First, micropeptides differ from conventional proteins in being very short (normally < 100 aa) and in having a physicochemical structure unsuitable for traditional mass spectrometry[15–17]. Second, micropeptides are commonly not conserved, and are thus young gene products, in terms of human evolution[17–19]. Third, most micropeptides are expressed at low levels, below the mass spectrometry threshold for peptide identification[20]. New methods are therefore urgently needed to identify these micropeptides, potentially using bioinformatics to predict lncRNAs that might encode peptides, followed by experimental verification.

## Prediction micropeptides by ORF, IRES, and m6A sites

An ORF is a theoretical aa-coding region, which is generally identified by analyzing the DNA nucleic acid sequence. ORF usually starts with ATG/AUG and extends to a stop codon [21]. Short ORFs are usually < 300 nucleotides long [22, 23]. ORF Finder and ORF Predictor are commonly used to search for ORFs in lncRNAs [24].

Regulatory elements such as IRES and m6A-modified conserved sites bind upstream in the ORF to mediate translation[25, 26]. IRES recruit ribosomes and initiate protein translation [27, 28]. LncRNAs with an IRES could translate a micropeptide on a continuous ORF [29, 30]. IRESite, IRESfinder, and IRESPred are currently used to predict the existence of IRES in lncRNAs[24]. The m6A modification plays essential roles in both mRNAs and lncRNAs, and is recently revealed to have important effects on the translation process. Various endogenous translatable lncRNAs may have m6A sites. Further studies of m6A have identified numerous predicted m6A sites, including M6APred-EL, M6AMRFS, SRAMP, and m6Acomet [24].

## Experimental verification of peptides

Most research on micropeptides has been based on Ribo-seq and RNC-seq [17, 24]. During translation, ribosomes bind and move along the mRNA chain to synthesize a peptide based on the codons, while forming a ribosome nascent chain complex [31]. Integrated Ribo-seq and RNC-seq analysis identified thousands of lncRNAs with the potential to encode polypeptide. In addition, Lu et al. [17] performed shotgun proteomics and detected 308 lncRNA-encoded peptides and further verified 207 unique peptides by peptide multiple reaction and parallel reaction monitoring.

Mass spectrometry (MS), as the current gold standard for protein detection, provides strong evidence for the existence of micropeptides[32]. However, as only 40% of lncRNA-encoded micropeptides are longer than 10 aa, many remain undetected[33]. Although graded MS can detect more putative micropeptides, it uses a reference database, which thus limits its ability to predict novel micropeptides [34]. Therefore, Cardon et al. [34] exploited a large-scale and unsupervised method based on cross-linking MS followed by shotgun proteomics to gather information on the functional role of novel isoforms and novel proteins (AltProts), mapping them with known pathways by identifying their reference protein (RefProts) interactors. In addition, OpenProt database not only annotates RefProts, but also AltProts. It also provides supporting evidence for each protein, such as MS, protein homology and predictions of functional domains [35]. MS is thus a powerful method for the discovery and verification of endogenously expressed micropeptides; however, although the presence of a micropeptide in the MS spectra strongly supports its existence, its absence from the spectra does not necessarily mean that it does not exist.

Adding Flag or GFP fusion proteins at the C-terminus before the stop codon is the usual approach for detecting micropeptides[36], and immunofluorescence detection of GFP expression or western blotting to detect specific Flag bands provides effective evidence for the existence of the peptides [37]. This evidence is enhanced by mutation of the start codon for GFP or the ORF [38], while preparation of specific monoclonal antibodies also confirms the existence of polypeptides and facilitates the subsequent search for proteins that interact with polypeptides [37, 38].

Chen et al. [39] developed a strategy that combines ribosome profiling, MS-based proteomics, and CRISPR-based screens to explore and characterize the widespread translation of functional micropeptides. Therefore, each method has specific advantages and disadvantages (Table 1), and combinations of Ribo-seq, RNC-seq, MS, and fusion proteins could provide more accurate results.

**Table 1:** Experimental verification of peptides

Techniques	Advantages	Disadvantages
Ribo-seq	Discover more potential peptides	Indirect evidence of translation, expensive, poor repeatability
RNC-seq	High sequence coverage and detection efficiency	Low sequencing accuracy, unable to obtain ribosome and ORF information
Mass spectrometry	Strong evidence, detect translation directly	Require specific expertise, rely on protein databases to search
Fusion protein	Easy to detect	May affect the structure and function of original protein

**Table 2:** Information of micropeptides encoded by lncRNAs in cancers

Numbers	LncRNA	Peptide	Size	Location	Conser-vative	Cancer types	Function
1	Linc00998	SMIM30	59aa	Membrane	Yes	Liver cancer	Promoting cell proliferation and migration
2	Loc90024	SRSP	130aa	Nucleus	Yes	Colon cancer	Promoting tumorigenesis and progression
3	Linc00908	ASPRS	60aa	Cytoplasm	ND	Breast cancer	Inhibiting angiogenesis
4	Linc00266	RBRP	71aa	Nucleus	No	Colon cancer	Promoting proliferation and metastasis
5	CASIMO1	SMIM22	10 kDa	Cytoplasm	Yes	Breast cancer	Affecting cell cycle, proliferation, cell motility
6	CRNDE	CRNDEP	84aa	Nucleus	Yes	Hela cell	Participating in cell proliferation and oxygen metabolism
7	HOXB-AS3	HOXB-AS3	53aa	Nucleus	Yes	Colon cancer	Inhibiting proliferation and tumor progression
8	MELOE	MELOE-3	54aa	ND	ND	Melanoma	Better T cell targets for immunotherapy
9	Linc01420	NoBody	7 kDa	Cytoplasm	Yes	Lung cancer	Localizing to P-body and resulting in P-body dispersal
10	UBAP1-AST6	UBAP1-AST6	12.8 kDa	Nucleus	ND	Lung cancer	Promoting cell proliferation and colony formation

*ND* No detection

LncRNA-encoded micropeptides have recently begun to attract widespread attention. Furthermore, increasing interest in micropeptides and improvements in sequencing technologies mean that more and more lncRNAs have been shown to have the potential to encode micropeptides, especially in cancers. In this review, we summarize the functional reported micropeptides encoded by lncRNAs in cancers. The new roles of lncRNAs may provide novel perspectives for cancer diagnosis and treatment.

## Tumor-related micropeptides encoded by lncRNAs

To date, lncRNAs have been shown to encode several functional micropeptides in various cancers. They are summarized as follows.

### SMIM30

**Fig. 1:**
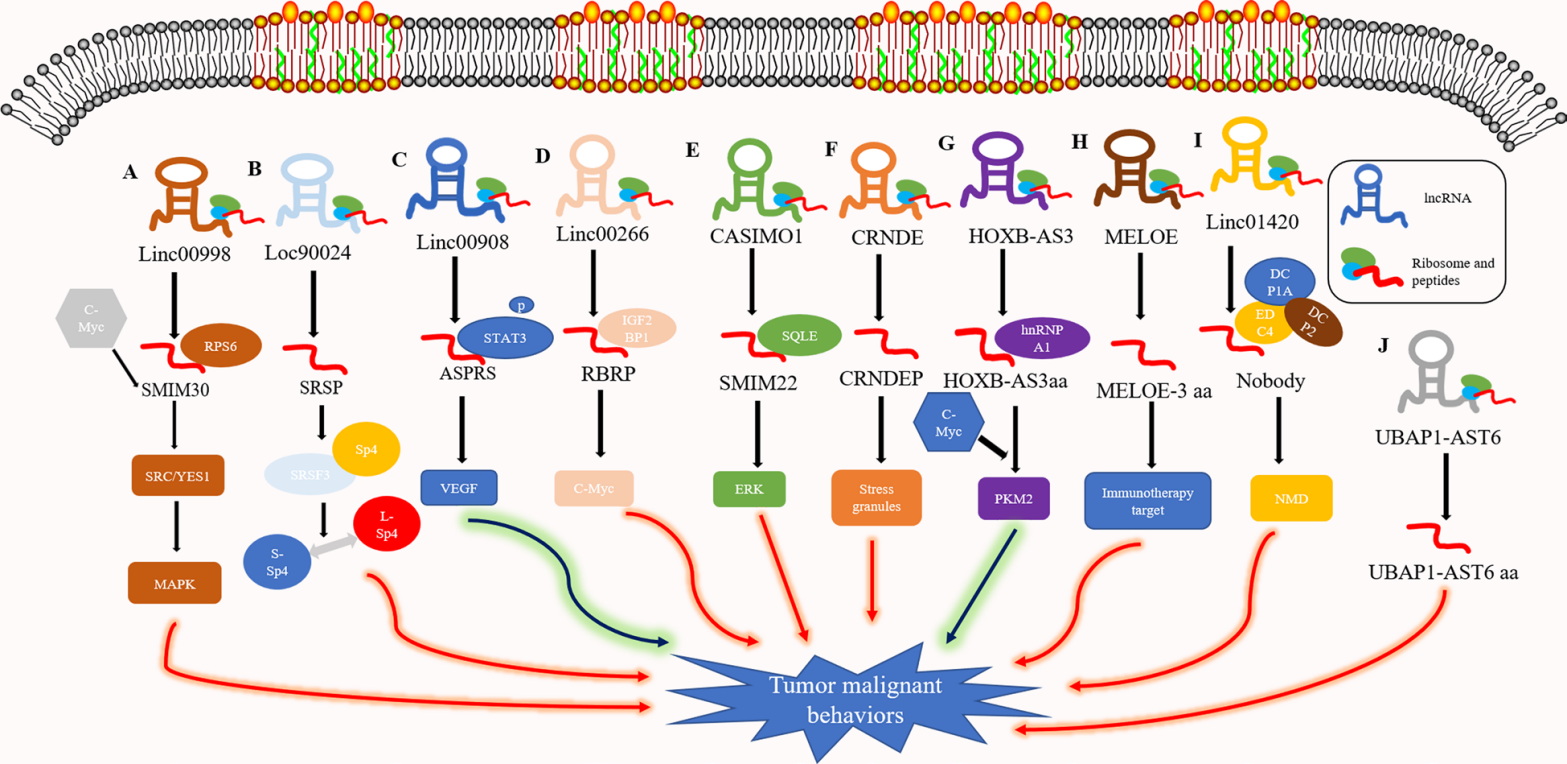
**Micropeptides encoded by lncRNAs regulate tumor malignant behaviors** **a** Linc00998 encodes micropeptide SMIM30 promotes HCC development by inducing SRC/YES1 membrane anchoring and MAPK pathway activation.** b** lncRNA LOC90024 encodes small protein SRSP induces "cancerous" Sp4 splicing variant formation.** c** Linc00908 encodes peptide ASPRS, which interacts directly with STAT3, thereby suppressing STAT3 phosphorylation. ASPRS also decreased VEGF levels and inhibited angiogenesis.** d** Linc00266 encodes RBRP peptide and RBRP interacts directly with IGF2BP1 thus promoting the mRNA stability of c-Myc by enhancing m6A recognition.** e** Micropeptide CASIMO1 are exerted via the SQLE protein and downstream ERK signaling pathway, thus affecting the cell metabolism equilibrium** f** LncRNA CRNDE encodes a peptide, CRNDEP, and the peptide promotes the formation of stress granules and affects cell proliferation and oxygen metabolism.** g** LncRNA HOXB-AS3 encodes the peptide HOXB-AS3 aa, which competitively binds hnRNP A1 and antagonizes hnRNP A1-mediated PKM splicing regulation.** h** IRES-dependent MELOE-3 aa antigens provide promising T cell targets for immunotherapy of melanoma.** i** Linc01420 encodes nobody, which binds EDC4 to regulate mRNA degradation. Linc01420 may promote nasopharyngeal carcinoma invasion and metastasis via this pathway.** j** LncRNA UBAP1-AST6 encodes UBAP1-AST6, which is a cancer-promoting factor in lung cancer cells.

Through RIP-seq assay of ribosomal protein S6 (RPS6), Pang et al. focused on one lncRNA, linc00998, with coding potential in hepatocellular carcinoma (HCC). The small endogenous peptide encoded by linc00998 was named SMIM30 (Fig. 1A, Table 2). They also explored the function and mechanism of the micropeptide in HCC. The results showed patients with higher levels of SMIM30 had a poorer survival rate. SMIM30, rather than the lncRNA itself, facilitated HCC tumorigenesis by regulating cell proliferation and migration. Moreover, c-Myc increased SMIM30 transcription and SMIM30 promoted the non-receptor tyrosine kinase SRC/YES1, thus activating the MAPK signaling pathway[40].

### SRSP

Yan’s team discovered lncRNA LOC90024 encoded a small 130 aa micropeptide found in colorectal cancer (CRC), termed SRSP (Fig. 1B, Table 2). High expression of SRSP was positively associated with malignant phenotypes and poor prognosis in CRC patients. SRSP, not LOC90024 itself, promoted CRC carcinogenesis and development. And downregulation of SRSP inhibited CRC progression. Mechanically, SRSP interacted with the RNA splicing regulator, SRSF3, to regulate mRNA splicing. SRSP promoted SRSF3 binding to transcription factor Sp4 exon 3, contributed to promoting the formation of the “oncogene” long Sp4 isoform, and restrained the formation of the “tumor suppressor” short Sp4 isoform. Overall, their findings revealed that the lncRNA-encoded micropeptide SRSP promoted “oncogene” Sp4 splicing variant formation. SRSP is a potential prognostic biomarker and therapeutic target for CRC patients[41].

### ASPRS

Linc00908 had been reported to be highly expressed in liver cancer and to interact with SOX4, thereby increasing its stability by inhibiting proteasomal degradation[42]. Linc00908 sponges with miR-483-5p in prostate cancer, and competitively reduces miR-483-5p targeting to *TSPYL5* (testis-specific Y-encoded-like protein 5) to exert its anticancer function[43]. A recent study reported on a small 60 aa regulatory micropeptide of STAT3 (ASPRS) encoded by linc00908 in patients with triple negative breast cancer (TNBC) (Fig. 1C, Table 2). The peptide is downregulated in TNBC and its expression is negatively related to tumor growth and overall survival. Molecular research revealed that estrogen receptor alpha (one of the three most common breast cancer markers) bound to the promoter region of linc00908 and regulated ASPRS expression. Furthermore, ASPRS interacts directly with the STAT3 CCD domain (important for STAT3 autophosphorylation), thereby suppressing STAT3 phosphorylation. ASPRS also decreased vascular endothelial growth factor (VEGF) levels and inhibited angiogenesis. In addition, VEGF expression is obviously higher in TNBC than non-TNBC tissues. These studies suggest that the ASPRS peptide functions as a tumor suppressor via the STAT3/VEGF signaling pathway, and may represent a potential therapeutic target in patients with TNBC [37].

### RBRP

Linc00266-1 was previously annotated as a lncRNA, but there were no relevant reports for it. However, Yang et al. predicted that linc00266 had the potential to code for a 71 aa polypeptide, referred to as RBRP (Fig. 1D, Table 2). RBRP is upregulated in CRC tissues and cells compared with controls, and high expression of RBRP is associated with a poor overall survival rate, and it acts as an independent prognostic factor in patients with CRC. *In vitro* and *in vivo* assays indicate that RBRP promoted CRC progression by affecting cell proliferation and metastasis. Further research demonstrated that RBRP interacted directly with the m6A reader insulin-like growth factor 2 mRNA-binding protein 1 (IGF2BP1) via a specific domain. RBRP, rather than linc00266-1, promoted the mRNA stability of the well-known oncogene c-Myc by enhancing m6A recognition on c-Myc mRNA via IGF2BP1. The study of the oncopeptide RBRP has thus revealed the diverse functions of lncRNAs and the close association between lncRNAs and m6A in carcinogenesis[36].

### CASIMO1

CASIMO1, also known as small integral membrane protein 22 (SMIM22), was previously incorrectly annotated as a lncRNA, prior to the discovery of a novel 10 kDa microprotein (Fig. 1E, Table 2). It is upregulated in estrogen receptor/progesterone receptor-positive compared with hormone-negative breast cancers. Furthermore, knockdown of CASIMO1 leads to G0/G1 cell cycle arrest and inhibition of cancer cell proliferation, and this inhibition was shown to be caused by CASIMO1, rather than by the lncRNA. Loss of CASIMO1 is associated with disruption of the actin cytoskeleton organization, resulting in attenuated cell motility. Regarding its mechanism, CASIMO1 positively regulates squalene epoxidase (SQLE) and its downstream extracellular signal-regulated kinase phosphorylation as CASIMO1 interacted with SQLE spatially. SQLE is a known oncogene product and an essential enzyme in cholesterol synthesis in breast cancer. Furthermore, knockdown of SQLE results in a similar phenotype to CASIMO1 downregulation, and overexpression of SQLE partly rescues the effect of CASIMO1-knockdown. These results suggest that the effects of the micropeptide CASIMO1 are exerted via the SQLE protein and downstream ERK signaling pathway, thus affecting the cell metabolism equilibrium[44].

### CRNDEP

Colorectal neoplasia differentially expressed (CRNDE) is a well-known lncRNA with key roles via various mechanisms in different kinds of cancers [45, 46]. For example, lncRNA CRNDE sponges miR-183 to regulate cyclin B1 (CCNB1) expression in cervical cancer [47], and also promotes the epithelial-mesenchymal transition(EMT) in liver cancer cells by activating the Wnt/β-catenin signaling pathway [48]. Herein, we introduced its unknown feature on the other side. LncRNA CRNDE encodes an 84 aa peptide, CRNDEP, predominantly located in the nucleus, which shows increased expression in rapidly proliferating tissues, such as the intestine and spermatocytes (Fig. 1F, Table 2). Downregulation of CRNDE lncRNA inhibits CRNDEP peptide expression. The peptide promotes the formation of stress granules and is localized to these granules. These preliminary results suggest that CRNDEP may participate in cell proliferation and oxygen metabolism. However, further studies are needed to elucidate the function and mechanism of CRNDEP [49].

### HOXB-AS3

LncRNA HOXB cluster antisense RNA 3 (HOXB-AS3) has previously been shown to act as an oncogene in various cancers[50]. For example, HOXB-AS3 promotes hepatocellular carcinoma by suppressing p53 expression[51], regulates ribosomal RNA transcription in NPM1-mutated acute myeloid leukemia [52], contributes to the malignant biological behavior of lung cancer via the phosphoinositide 3-kinase/Akt pathway [50], and exacerbates ovarian cancer progression via the Wnt/β-catenin signaling pathway [53].

In a CRC study, HOXB-AS3 was shown to encode a 53 aa micropeptide, (Fig. 1G, Table 2) having previously been wrongly annotated as a noncoding RNA because it was found bound to ribosomes in a ribosome profiling study [54]. LncRNA HOXB-AS3 is downregulated in CRC tissues, especially in highly metastatic cancer cells. HOXB-AS3 peptide expression is also reduced in CRC, and Kaplan-Meier survival curve analysis indicated that lower expression levels of HOXB-AS3 peptide were associated with a poorer prognosis. Knockdown of lncRNA HOXB-AS3 decreases peptide levels and inhibits CRC proliferation and tumor progression in a series of *in vivo* and *in vitro* experiments; however, the peptide, rather than its lncRNA, has been shown to be responsible for its key role in suppressing cancer.

The molecular mechanism of the HOXB-AS3 peptide in CRC development has been characterized by identifying the proteins interacting with the peptide. Among these proteins, heterogeneous nuclear ribonucleoprotein A1 (hnRNPA1) modulates pyruvate kinase M (PKM) splicing, thereby affecting cancer metabolic reprogramming, such as aerobic glycolysis, and was therefore selected for further study. Downregulation of hnRNPA1 mimicks the effects of overexpression of HOXB-AS3 peptide, and re-expression of hnRNPA1 in HOXB-AS3 peptide-overexpressing cells rescues the anticancer effect. Mechanistically, HOXB-AS3 peptide competitively blocks the binding of hnRNPA1 to PKM by binding to the arginine residues in the RGG motif of hnRNPA1, thus inhibiting PKM2 expression and glucose metabolism [38].

### MELOE

Researchers demonstrated that the lncRNA MELOE produced three polypeptides, MELOE-1, MELOE-2, and MELOE-3, of 39, 44, and 54 aa long, respectively [55–57]. MELOE RNA was shown to be polycistronic, and translation of MELOE-1 and MELOE-2 depends on internal ribosome entry site (IRES) sequences [58], while MELOE-3 is translated via a classical cap-dependent mechanism. Furthermore, expression levels of MELOE-1 and MELOE-2 decreased in melanoma and were undetectable in melanocytes, while MELOE-3 was highly expressed in melanoma and melanocytes. MELOE3 also shows poor immunogenicity and immune tolerance, while MELOE-1 and MELOE-2 are highly specific antigens in melanoma, with relevance for T cell immunosurveillance (Fig. 1H, Table 2). In summary, IRES-dependent MELOE family polypeptide antigens provide promising T cell targets for immunotherapy of melanoma [57].

## NoBody

Linc01420 is upregulated in nasopharyngeal cancer and is positively associated with tumor metastasis and an unfavorable prognosis[59]. Linc01420 also facilitates cell proliferation and migration in melanoma [60], promotes pancreatic cancer development via targeting KRAS [61], and accelerates cell cycle progression and cell proliferation in thyroid cancer [62]. Proteomics analysis showed that the 7 kDa micropeptide, non-annotated P-body dissociating micropeptide (NoBody), was translated from LINC01420/LOC550643 in leukemia K562 and breast cancer MDA-MB-231 cells (Fig. 1I, Table 2). The biological function of NoBody has been explored by co-immunoprecipitation and mass spectrometry, which revealed enrichment of decapping complex proteins, which removes the 5ʹ cap from mRNA and thus promotes 5ʹ-3ʹ decay and participates in mRNA turnover and nonsense-mediated decay. Among these proteins, enhancer of decapping proteins 4 (EDC4) is the most abundant. The micropeptide mainly localizes to P-bodies (decapping degradation of mRNA at the 5ʹ end and is mainly carried out in P-bodies in the cytoplasm), resulting in P-body dispersal rather than degradation. NoBody expression is thus negatively associated with P-body numbers by directly interacting with EDC4. Nevertheless, the influence of NoBody on cancer growth, metabolism, and progression remains to be clarified [63].

### UBAP1-AST6

Ribo-seq and ribosome nascent chain complex sequencing (RNC-seq) were carried out to investigate lncRNAs that might encode micropeptides, and identified thousands of lncRNAs bound to ribosomes with putative protein-encoding capabilities. Based on laboratory evidence (mass spectrometry, bioinformatics, antibodies), > 300 proteins encoded by lncRNAs were verified, including UBAP1-AST6, which is widely present in human cell lines (lung cancer and hepatic carcinoma) and tissues (joint, placenta, and prepuce). Subsequent research showed that this micropeptide was located in the nucleus (Fig. 1J, Table 2). Moreover, UBAP1-AST6 promoted A549 cell proliferation and colony formation, and rescue assay confirmed the function of UBAP1-AST6 in lung cancer cells[17].

## Other functional lncRNA-encoded micropeptides

In addition to directly participating in tumorigenesis, lncRNA-encoded micropeptides also exert important effects in inflammation, metabolism, and signal transduction pathways, which are also closely associated with cancer.

## Metabolism

Linc00116 encodes a 56 aa peptide, Mtln, which is localized in mitochondria. Mtln interacts with NADH-dependent cytochrome b5 reductase and disrupts its mitochondrial localization, thereby increased oxygen consumption and respiratory complex I activity [64]. Consistent with this, another study also revealed that Mtln promoted Ca^2+^ buffering ability and mitochondrial respiration while inhibiting reactive oxygen species, thus enhancing respiratory efficiency [65].

## Inflammation

Inflammation-modulating micropeptide (IMP) is a 44 aa micropeptide encoded by an unrecognized ORF of lncVLDLR. IMP was shown to be highly homologous to transcription factors related to inflammatory immune response factors, such as nuclear factor-κB. Overexpression of IMP in THP1 macrophages induces chemokine and cytokines levels, suggesting that it is involved in an inflammatory response by interacting with transcriptional coactivators [66].

## Signaling

Micropeptides also participate in signaling pathways. For example, stress- and tumor necrosis factor (TNF)-α-activated ORF micropeptide (STORM) derived from linc00689 is actuated by TNF-α-induced and mammalian ste20-like kinase mediated phosphorylation of translation initiation factor eIF4E [67]. In addition, the micropeptide Toddler accelerates gastrulation by activating APJ/Apelin receptor signaling[68].

## Future perspectives of micropeptides

New cancer treatments, such as immunotherapy and targeted therapy, have emerged in recent years, and their combinations with traditional surgery, radiotherapy, and chemotherapy have greatly improved the prognosis of some cancer patients; however, the overall survival rate for most patients remains poor[69, 70]. The health hazards and huge social burden associated with cancer have stimulated extensive research. Cancer-related lncRNAs are currently a hot research topic, especially in relation to lncRNA-encoded micropeptides. LncRNAs have been reported to be involved in carcinogenesis and tumor development in various ways, and the increasing role of lncRNA-encoded micropeptides has attracted a great deal of attention. Research has confirmed the existence and importance of lncRNA-encoded functional micropeptides. However, it is still difficult to assess lncRNA coding potential as the database used to predict the conservation of ORFs, IRES sequences, and m6A sites in lncRNAs is incomplete, and experimental validation approaches are still immature. Therefore, the actual number of micropeptides and their potential biological functions remain unclear.

In this paper, we reviewed the current literature on cancer-related lncRNA encoded-micropeptides and other classic peptides that are associated with inflammation, metabolism, and signal transduction. These studies provided novel perspectives on lncRNA biological functions and molecular mechanisms. Among them, ASPRS and HOXB-AS3 are tumor suppressors while RBRP, CASIMO1, CRNDEP, NoBody, UBAP1-AST6, and MELOE are defined as oncogenes. Similar to lncRNAs or coding genes, micropeptides are distributed in the cytoplasm and bind to specific proteins involved in signaling pathways [37, 44], or may be concentrated in the nucleus to impact mRNA stability [36] or kinase splicing [38]. Some of these micropeptides are conserved[38, 44, 49, 71], while many undiscovered micropeptides are likely to be non-conservative because they are the products of young genes. This indirectly suggests that known tumor-associated lncRNA-encoded conservative micropeptides are not produced by young genes in terms of human evolution, and these conserved micropeptides are likely to play an irreplaceable role in the biological process. As for the currently reported tumor-related lncRNA-encoded micropeptides, some are conserved, probably because conservation may reflect biological importance. But the majority of micropeptides are not conserved and it is not clear whether they have any biological function as they may be rapidly degraded after translation.

Indeed, in addition to lncRNA-encoded micropeptides, circRNAs and pri-miRNAs may also encode functional micropeptides[72, 73]. Micropeptides thus represent a promising target for cancer drug research or as biomarkers for prognosis prediction.

Medical research advocates the use of precision medicine to reduce side effects and drug resistance caused by traditional chemotherapy. Compared with chemotherapeutic drugs, micropeptide agents have advantages including high specificity and activity, less cytotoxicity, and low immunogenicity (39). Moreover, there is a precedent for using micropeptides to treat cancer, such as the use of mifamurtide (a synthetic lipophilic muramyl dipeptide analogue) for osteosarcoma [74]. However, although the use of micropeptides for cancer treatment is currently under investigation, no researchers have yet put the idea into practice, and despite its potential as a target for cancer therapy, certain issues need to be fully considered. Micropeptides may have a very short half-life and their ability to reach the tumor site and enter the tumor cells via transporters on the cell membrane remain to be clarified. Nanoscale carriers can be used as a strategy for improving cancer treatments, by enhancing the accumulation and prolonging the action time of anti-cancer drugs. If these micropeptides thus have difficulty reaching the tumor cells or if their half-life is too short, they could potentially be wrapped in nanomaterials (overexpressed virus for tumor suppressor or small interfering RNA for oncopeptide) to avoid their rapid metabolism and facilitate their successful delivery to the designated location to exert their anti-cancer effect.

In addition, although the differential expression and prognostic correlation of several micropeptides have been confirmed by western blot and immunohistochemistry, no ncRNA-encoded micropeptides have been detected in body fluids. If micropeptides that are highly expressed in tumor tissues could be detected in body fluids, they could provide useful biological markers for detecting therapeutic effects, tumor recurrence, and prognosis.

## Conclusions

In conclusion, lncRNA-encoded micropeptides open up new horizons and provide a new hot topic for future research into cancer drugs and biomarkers. Micropeptides enrich and broaden the diversity of roles of lncRNAs in cancer development. The micropeptides discussed in this review affect tumors in various ways. However, the lncRNA-encoded micropeptides that have been discovered so far are only the beginning. More micropeptides and underlying mechanisms need to be explored. We believe that more functional micropeptides encoded by lncRNAs, which have been overlooked in gene annotations, will be characterized in the future. And they will provide many opportunities for developing cancer biomarkers, drug targets, and small molecule peptide drugs.

## Data Availability

Not applicable.

## Abbreviations

LncRNA: Long noncoding RNA
circRNAs: Circular RNAs
snoRNAs: Small nucleolar RNAs
m6A: N6-methyladenosine
BMP: Bone morphogenetic protein
ORF: Open reading frame
RPS6: Ribosomal protein S6
HCC: Hepatocellular carcinoma
CRC: Colorectal cancer
ASPRS: A small regulatory peptide of STAT3
TSPYL5: Testis-specific Y-encoded-like protein 5
TNBC: Triple negative breast cancer
VEGF: Vascular endothelial growth factor
RBRP: RNA binding regulatory peptide
IGF2BP1: Insulin-like growth factor 2 mRNA-binding protein 1
CASIMO1: Cancer-associated small integral membrane open reading frame 1
SMIM22: Small integral membrane protein 22
SQLE: Squalene epoxidase
CRNDE: Colorectal neoplasia differentially expressed
HOXB-AS3: HOXB cluster antisense RNA 3
HNRNPA1: Heterogeneous nuclear ribonucleoprotein A1
PKM: Pyruvate kinase M
IRES: Internal ribosome entry site
NoBody: Non-annotated P-body dissociating micropeptide
EDC4: Enhancer of decapping proteins 4
Ribo-seq: Ribosome profiling
RNC-seq: Ribosome nascent chain complex sequence
aa: Amino acid
CCNB1: Cyclin B1
EMT: Epithelial-mesenchymal transition
SPAR: Small regulatory polypeptide of amino acid response
SR: Sarcoplasmic reticulum
DWORF: Dwarf open reading frame
IMP: Inflammation-modulating micropeptide
STORM: Stress- and TNF-α-activated ORF micropeptide
MS: Mass spectrometry
